# Antimicrobial Resistance and Genomic Characterization of *Salmonella* Infantis from Different Sources

**DOI:** 10.3390/ijms24065492

**Published:** 2023-03-13

**Authors:** Angela Michela Immacolata Montone, Anna Cutarelli, Maria Francesca Peruzy, Immacolata La Tela, Roberta Brunetti, Maria Gerarda Pirofalo, Veronica Folliero, Anna Balestrieri, Nicoletta Murru, Federico Capuano

**Affiliations:** 1 Department of Food Inspection, Istituto Zooprofilattico Sperimentale del Mezzogiorno, Via Salute 2, 80055 Portici, Italy; 2 Department of Veterinary Medicine and Animal Production, University of Naples Federico II, Via Delpino 1, 80137 Naples, Italy; 3 Salmonella Typing Centre of the Campania Region-Department of Food Microbiology, Istituto Zooprofilattico Sperimentale del Mezzogiorno, Via Salute 2, 80055 Portici, Italy; 4 Epidemiology and Biostatistics Coordination Department, Experimental Zooprophylactic Institute of Southern, Via Salute 2, 80055 Portici, Italy; 5 Complex Operative Unit of Pathology and Microbiology, Microbiology Sector, University Hospital San Giovanni di Dio e Ruggi d’ Aragona, Largo Città di Ippocrate, 84131 Salerno, Italy; 6 Department of Experimental Medicine, University of Campania “Luigi Vanvitelli”, 80138 Naples, Italy; 7 Task Force on Microbiome Studies, University of Naples Federico II, 80138 Naples, Italy

**Keywords:** *S*. Infantis, antimicrobial resistance, poultry, MLVA

## Abstract

The epidemiology of Salmonella Infantis is complex in terms of its distribution and transmission. The continuous collection and analysis of updated data on the prevalence and antimicrobic resistance are essential. The present work aimed to investigate the antimicrobial resistance and the correlation among *S.* Infantis isolates from different sources through the multiple-locus variable-number of tandem repeat (VNTR) analysis (MLVA). A total of 562 Salmonella strains isolated from 2018 to 2020 from poultry, humans, swine, water buffalo, mussels, cattle, and wild boar were serotyped, and 185 *S.* Infantis strains (32.92%) were identified. *S.* Infantis was commonly isolated in poultry and, to a lesser extent, in other sources. The isolates were tested against 12 antimicrobials, and a high prevalence of resistant strains was recorded. *S.* Infantis showed high resistance against fluoroquinolones, ampicillin, and tetracycline, which are commonly used in human and veterinary medicine. From all *S.* Infantis isolates, five VNTR loci were amplified. The use of MLVA was not sufficient to understand the complexity of the epidemiological relationships between *S.* Infantis strains. In conclusion, an alternative methodology to investigate genetic similarities and differences among *S.* Infantis strains is needed.

## 1. Introduction

The European Food Safety Authority (EFSA) estimates that salmonellosis results in a yearly economic burden of three billion euros in the European Union [1]. In Italy, the disease is the leading cause of foodborne infections, with over 3500 cases reported annually [2].

The genus *Salmonella* consists of two species: *Salmonella enterica* and *Salmonella bongori*. *Salmonella enterica* (*S*) is further divided into six subspecies that include over 2600 serotypes [3]. Based on their different pathogenic behaviors and disease manifestation, *Salmonella* serotypes can be classified into typhoidal: highly adapted to humans and higher primates, and non-typhoidal (NTS): for which the gastrointestinal tract of a wide range of domestic and wild animals is regarded as the reservoir [4]. Worldwide, serovars belonging to the NTS group play a significant role in human salmonellosis, and the transmission among humans and animals mainly occurs through the direct contact or the ingestion of contaminated foods, such as eggs, poultry, fish, eggs, beef, and dairy products. In recent years, the most isolated serovar from food sources and animals worldwide has been *S.* Infantis, especially from poultry and poultry products [5].

In the EU, the implementation of national control programs for *Salmonella* in accordance with Reg. (EC) No 2160/2003 has led to a steady reduction of Salmonella infections in poultry over the last two decades. Since 2003, in *Gallus gallus* breeding flocks, the serovars considered relevant for human health and subject to control under the legislation are *S.* Enteritidis, *S.* Typhimurium (including the monophasic variant), *S.* Infantis, *S.* Hadar, and *S.* Virchow. However, restrictive measures in the case of the identification of *S.* Infantis have been implemented only since 2019 [6]. Moreover, in poultry, the vaccination has been administered only against *S.* Enteritidis and *S.* Typhimurium. Over the years, the specific measures put in place against *S.* Enteritidis and *S.* Typhimurium have likely led to a massive spread of other serovars, and *S*. Infantis has been probably the most advantaged one [7]. In recent years, this serovar has also become a relevant agent of human salmonellosis [8,9] and is steadily the fourth most commonly detected *Salmonella* serovar in human cases of salmonellosis in Europe [10] with a stable prevalence in the last years (around 2.3%).

Human salmonellosis is usually characterized by self-limiting gastroenteritis and does not require antimicrobial treatment [11,12]. However, the infection can be more serious, and the use of antimicrobial agents such as fluoroquinolones and third-generation cephalosporins is recommended. *S.* Infantis, as well as other *Salmonella* serovars, can exhibit resistance to a wide range of antibiotics, including praised antibiotics [8]. Antibiotic resistance (AR) plays an important role in the increased incidence of different bacterial infections. Indeed, the high level of resistance and multi-drug resistance (MDR) recorded in *S.* Infantis in multiple countries (i.e., Italy, Hungary, Germany, Russia, United States) can be considered another reason for the epidemiological success of this serovar [13,14]. The extended AR exhibited by *Salmonella* species and other pathogenic bacteria is due to the use and misuse of antibiotics in humans and animals (both in livestock and aquaculture species); these latter, moreover, may spread antibiotic-resistant bacteria (ARB) via their faces and contaminate the terrestrial and marine environment [12]. According to the EU’s joint inter-agency antimicrobial consumption and resistance analysis (JIACRA) reports 2016–18 [15], the resistance in humans is linked to either antibiotic use in animals or the spread of resistant bacteria from animals to humans, rather than resistance in humans and antibiotic use in humans. In this context, it is essential to collect and analyze data on AR and to investigate transmission routes to implement specific action plans. Among the several fingerprinting methods used over the years for the evaluation of transmission routes, pulsed-field gel electrophoresis (PFGE) is still considered the gold-standard method [16]. However, the multiple-locus variable-number of tandem repeat (VNTR) analysis (MLVA) has emerged as an effective tool for the investigation of related strains with a discriminatory power higher than that of PFGE and easier to perform than other methods, such as the whole genome sequencing (WGS) [17]. However, data on the discriminatory power of this method on *S.* Infantis strains are still limited.

The aim of the present study was, therefore, to evaluate the antimicrobial resistance and the correlation among *S.* Infantis strains isolated from humans, animals, and food, through the application of MLVA.

## 2. Results

Out of the 562 *Salmonella* strains overall typed in the three-year study period, 185 (32.92%) belonged to the serotype Infantis (antigenic formula 6,7:r:1,5), of which 162 strains (162/176, 92.05%) were isolated from poultry-related samples, seven strains isolated from mussels (7/48, 14.58%), seven strains isolated from humans (7/130, 5.38%), three strains isolated from swine (3/96, 3.13%), two strains isolated from cattle (2/30, 6.67%), two strains isolated from water buffalo (2/72, 2.78%), and two isolated wild boars (2/10, 20.00%) (Table 1).

Among the strains isolated from poultry, the highest percentage of *S*. Infantis was isolated from poultry products (n. 78, 48.15%) (Table 1).

The difference in the isolation of *S*. Infantis from samples of poultry origin to all other sources was significant (*Z from 10.73 to 15.07*), as was the comparison between the poultry isolates and all the other sources together (*Z = 20.82*).

### 2.1. Minimal Inhibitory Concentration

Out of the 185 *S.* Infantis strains analyzed, 12 (6.5%) showed susceptibility to all antibiotics tested. Overall, 75.13% (n. 139) were resistant to at least four antibiotics. In particular, one strain isolated from poultry meat showed resistance to eleven antibiotics.

**Figure 1:**
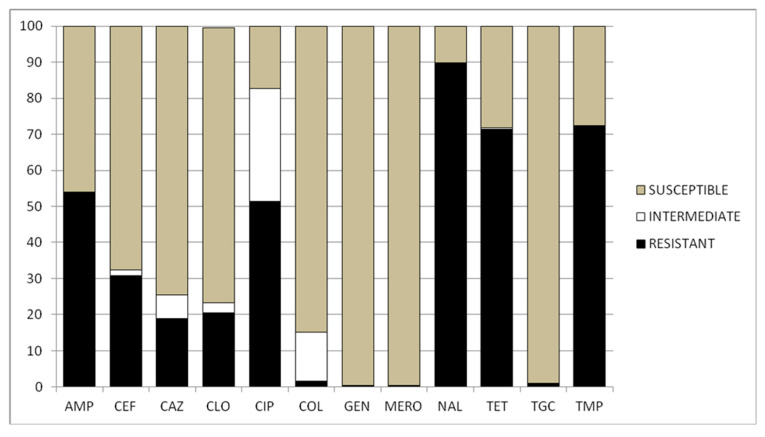
Overall occurrence (%) of susceptibility, intermediate resistance, and resistance to Ampicillin (AMP), Cefotaxime (CEF), Ceftazidime (CAZ), Chloramphenicol (CLO), Ciprofloxacin (CIP), Colistin (COL), Gentamicin (GEN), Meropenem (MERO), Nalidixic acid (NAL), Tetracycline (TET), Tigecycline (TGC), and Trimethoprim (TMP) in the *S.* Infantis serotypes.

High proportions of *Salmonella* isolates were resistant to nalidixic acid (n. 166, 89.7%), trimethoprim (n = 134, 72.4%), tetracycline (n. 132, 71.3%), and ampicillin (n. 100, 54.0%). However, considering the strains that displayed intermediate resistance as resistant, *S.* Infantis exhibited a very high resistance to ciprofloxacin as well (n. 153, 82.70%) (Figure 1).

**Figure 2:**
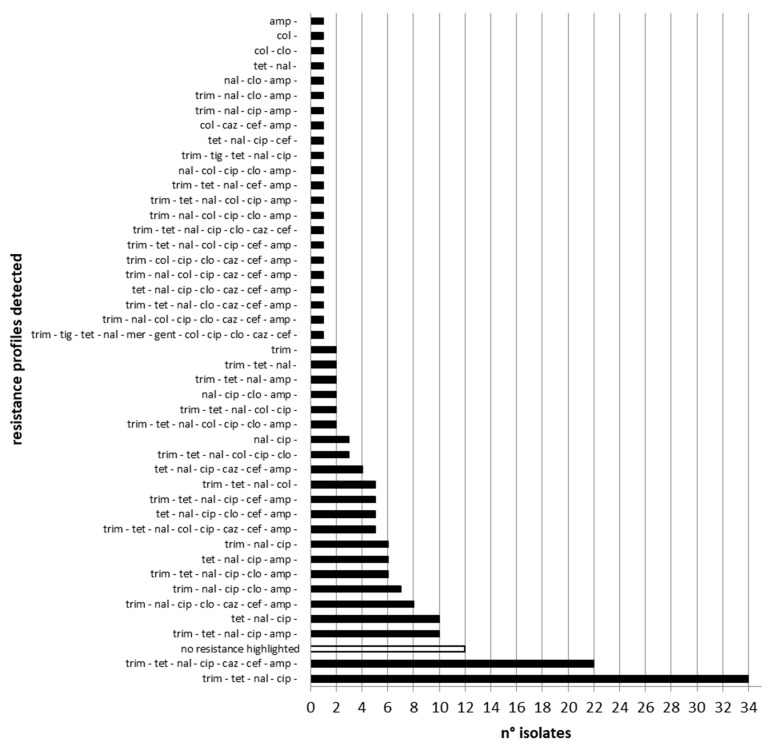
Occurrence (n.) of resistance profiles observed in *S.* Infantis strains isolated from 2018 to 2020.

Moreover, the 185 strains analyzed showed 44 different patterns of resistance (Figure 2). Overall, 162 strains (87.57%) showed co-resistance to (fluoro)quinolones (nalidixic acid and ciprofloxacin) and 47 strains (25.00%) to both cephalosporins tested. Moreover, 45 strains (24.32%) showed co-resistance to all fluoroquinolones and third-generation cephalosporins tested. In total, 148 (80.00%) *S.* Infantis isolates were classified as multidrug-resistant (MDR).

In relation to source, only two strains of poultry origin exhibited sensitivity to all antibiotics, whereas 142 (87.65%) were MDR and 139 (85.80%) were resistant to at least four molecules. *S.* Infantis strains isolated from poultry were highly resistant to almost all antibiotics tested and were particularly resistant to nalidixic acid (157, 96.91%) (Table 2). Among strains isolated from humans, five out of seven were resistant to at least four antibiotics, including ampicillin, ciprofloxacin, nalidixic acid, and tetracycline.

Low levels of resistance were observed in strains isolated from mussels and other mammals (cow/calves, water buffalo, wild boars, and pigs) (Table 2). The lower susceptibility of poultry strains compared to those from other sources was statistically significant (X^2^ = 74; *p* < 0.05,OR = 147).

### 2.2. MLVA

**Figure 3:**
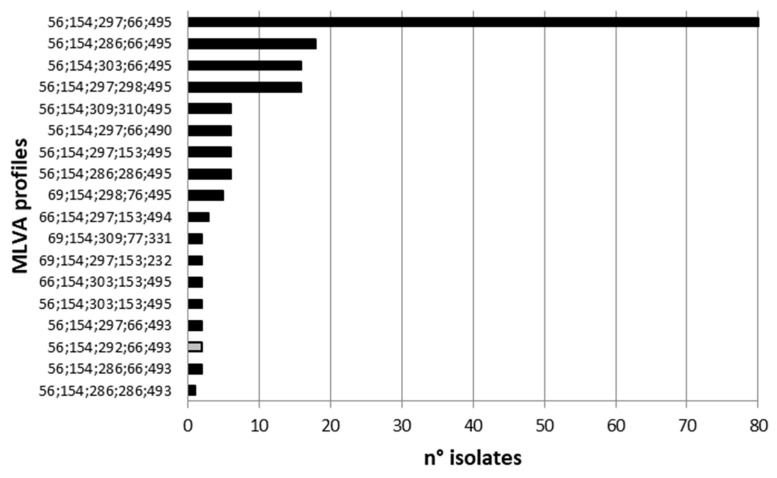
Occurrence (n.) of MLVA profiles observed in *S.* Infantis strains isolated from 2018 to 2020.

Out of 185 isolates, 18 distinct MLVA profiles (genotypes) were identified. The most common MLVA profile, 56-154-297-66-495, accounted for 43.2% (80 isolates) of the isolates. The remaining 17 profiles included 2 to 17 isolates each (Figure 3).

All isolates were amplified using the loci selected in this study. The locus STTR9 was detected as a single allele, while the locus SG2 showed six different alleles, STTR3 showed five alleles, STTR5 showed four alleles, and Sty19 showed three alleles. (Table 3).

The genetic diversity based on allele discriminatory power (ADP) of the five considered loci ranged from 0.0 to 0.55 (Table 3). VNTR SG2 and STTR5 were the most polymorphic loci (ADP 0.55 and 0.53, respectively), Sty19 and STTR3 were less polymorphic (ADP 0.24 and 0.25 respectively), while STTR9 lacked polymorphism and discriminating power (Table 3).

The clustering of MLVA profiles revealed the presence of six major clusters (Cluster 1 = 19 strains, Cluster 2 = 98 strains, Cluster 3 = 23 strains, Cluster 4 = 27 strains, Cluster 5 = 6 strains, and Cluster 6 = 12 strains). The dendrogram is reported in the Supplementary Material (Figure S1).

MLVA cluster 1 consisted mainly of the profile 56;154;286;66;495 (78.95%), cluster 2 mainly of 56;154;297;66;495 (82.98%), cluster 3 mainly of 56;154;303;66;495 (30.43%), cluster 4 mainly of 56;154;297;298;495 (29.63%), cluster 5 mainly of 69;154;309;77;331 (50.00%) cluster 6 mainly of 69;154;309;66;495 (33.33%).

Few correlations (genetic similarity) between *S.* Infantis strains belonging to the same cluster were recorded by analyzing the geographical location, antibiotic resistance profile, source, and date of sampling (Table 4).

## 3. Discussion

The choice to study *S.* Infantis in this research was due to its high occurrence in southern Italy [7]. From 2018 to 2020, out of 562 Salmonella isolates collected in the Campania and Calabria regions from humans, animals, and food, 185 were identified as *S.* Infantis.

As previously reported [18], *S*. Infantis is the most prevalent serovar in poultry, with 92.05% of the strains belonging to this serovar. In recent years in Europe, human infections caused by *S.* Infantis have almost doubled, and most of these infections were associated with broiler origin [18]. In the present study, the isolation frequency of this serovar in the Campania and Calabria regions (5.38% of the total cases) was higher compared with those reported in Europe by the EFSA in the same period (2018 = 2.3%, 2019 = 2.4%, 2020 = 2.5%) [19].

In regards to animal sources other than poultry, the highest percentage of strains belonged to serovars other than *S.* Infantis. Indeed, according to the literature, the most commonly reported serovars in swine are *S*. Derby and Typhimurium [4], whilst in cattle, *S.* Enteritidis and *S.* Schleissheim were reported as the dominant serovars in slaughtered cattle, and *S.* Dublin in beef in a study conducted in Poland [20], *S*. Typhimurium, *S.* Enteritidis, and *S.* Newport in Cattle were reported as the dominant serovars in a study conducted in Turkey [21], *S.* Enteritidis, *S.* Cholerasuis, *S*. Typhimurium and *S.* Pullorom in raw beef in a study conducted in Pakistan [22] and *S*. Typhimurium and *S.* Stanley in bovine meet and carcasses in an Italian study [7]. To our knowledge, studies on the *Salmonella* serovars distribution in water buffalo are limited. However, the results of the present work are in contrast with those of Peruzy et al. (2022) [7], in which this serovar was never detected. However, the differences between the current study and the study of Peruzy et al. (2022) [7] may arise from the different sample types, since the bacterium in the study of Peruzy et al. was searched for on carcass surfaces. A high level of prevalence of *S.* Infantis was also recorded in mussels which, due to their filter-feeding activity, may concentrate *Salmonella* serovars introduced to aquatic environments via animal and human waste [23]. In regards to humans, the highest percentage of strains belonged to serovars other than *S.* Infantis. However, the percentage of *S.* Infantis reported in the present work (5.38%) was higher than the EU average [19].

In the present study, the antimicrobial resistance of S. Infantis isolated from different sources was tested against 12 antimicrobials, and 44 different patterns of resistance were recorded, confirming the wide diversity of resistance profiles in *S.* Infantis strains. A total of 173 bacterial strains (93.51%) proved resistant to at least one antibiotic.

The study results on the antimicrobial resistance of *S.* Infantis isolated from different sources match with the findings reported by the EFSA [24] for *Salmonella* spp. isolates in humans and/or animals. The highest levels of resistance were observed against nalidixic acid, trimethoprim, tetracycline, ampicillin, and ciprofloxacin, in accordance with previously reported results in Italy [12].

The resistances observed in the present study are of particular concern since fluoroquinolones (nalidixic acid and ciprofloxacin) represent the gold standard for treatment against invasive salmonellosis in humans, and ampicillin and tetracycline are widely used in veterinary medicine as first-line treatment in animal infections (Regulation (EU) 2019/6). The latter ones are, along with sulphonamides, the most commonly purchased antimicrobials for veterinary use [14].

A rate of 87.57% of *S*. Infantis strains showed a co-resistance to each fluoroquinolone (87.57%) and cephalosporins (25.00%) tested, while 24.32% exhibited resistance to both antibiotic classes.

Fluoroquinolones and third-generation cephalosporins are categorized as the highest priority critically important antimicrobials (CIA) in human medicine due to the limited availability of alternatives for the treatment of bacterial infections [25]. Moreover, the importance of the result of the present work lies in the fact that third-generation cephalosporins are used to treat human infections when fluoroquinolones are not recommended (e.g., during childhood infection).

Although the number of humans strains was limited, high levels of resistance were reported against nalidixic acid (71.43%), tetracycline (71.43%), ampicillin (71.43%) and ciprofloxacin (71.43%). Interestingly, for *S.* Infantis, the level of resistance to these antibiotics was higher than the European average, especially for ampicillin (17.4%). The latter differences may be attributed to the fact that Italy is one of the largest consumers of antimicrobials in the EU [14].

The study found that most of the 162 *S.* Infantis strains isolated from poultry were resistant to various antibiotics, including nalidixic acid (96.91%), ceftazidime (93.83%), ciprofloxacin (88.88%), trimethoprim (77.78%), and tetracycline (76.54%). These results align with the European findings reported by the EFSA [24]. Before 2022, group antibiotic treatments were common in poultry farming. However, in 2022, the regulation on veterinary medicines banned the routine use of antibiotics in farming, including group treatments [26]. The impact of this ban on antibiotic resistance (AR) remains to be evaluated through further research.

Interestingly, the percentage of resistance toward chloramphenicol recorded in the present study (resistant strains = 45.68%; strains with intermediate resistance = 3.09%) is alarming since the use of this compound is banned in food-producing animals in all the member states of the European Union. Although this is speculative, these results could be explained by the illegal and fraudulent use of this antimicrobial in veterinary practices [12].

The present study used multilocus variable-number tandem repeat analysis (MLVA) to examine the genetic diversity of *S*. Infantis strains. Studies using MLVA for discrimination of *Salmonella* enterica serovar Infantis are limited. This study used fragment lengths instead of tandem repeat numbers to describe allelic variation as determined by MLVA. Out of the 185 strains tested, 18 different MLVA profiles were identified, with the SG2 and STTR3 loci having the highest number of alleles. A previous study [9] proposed a 13-locus MLVA scheme for genotyping but found that its discriminatory power was inferior to Pulse-Field Gel Electrophoresis (PFGE) and Multiple-Locus Variable-Number Tandem Repeat Analysis (MAPLT). However, another study (Ranjbar et al., 2016) showed that MLVA had a higher discriminatory power. After cluster analysis, the isolates were divided into six clusters, but it was possible to correlate only some isolates included in the same cluster.

In the present study, the MLVA profile did not provide further clarity and was not a useful tool for epidemiological investigation. The use of the AMR profile and the MLVA profile, alone or in combination, was not sufficient to understand the complexity of the epidemiological relationships between locations within different production systems. Despite the high level of apparent diversity, cluster analysis was unable to differentiate the transmission pathways of all detected *S.* infantis isolates. This complexity cannot be resolved in the absence of intensive sampling programs for all generations of the production system [27]. Therefore, further studies should be performed to understand the complexity of the epidemiological relationships between *S.* Infantis strains.

## 4. Materials and Methods

### 4.1. Detection and Serotyping

From 2018 to 2020, a total of 562 *Salmonella* isolates were collected and analyzed by the Salmonella Serotyping Laboratory of the Campania Region (SSLCR). The strains originated from poultry (fresh meat, meat preparations, and eggs) (n. 176), human (fecal swabs, urine, and blood) (n. 130), swine (sponge) (n. 96), water buffalo (fecal swabs) (n. 72), mussels (farms and retail market) (n. 48); from cattle (meat and meat products) (n. 30) and wild boars (tissue/sponge) (n. 10). *Salmonella* isolates were collected from laboratories operating in the field of food control and animal diagnosis and from hospitals in the Campania regions (human strains). All human and animal isolates were from clinical cases. 

Serotyping was performed according to the Kauffman–White Le Minor scheme [28] by agglutination with specific anti-sera, executed through rapid assay on the slide for somatic antigens (Statens Serum Institute, Copenaghen, Danmark) and in-tube agglutination for the identification of flagellar antigens (Difco, Franklin Lakes, New Jersey, USA) as previously described [29].

### 4.2. Minimal Inibitory Concentration

The minimal inhibitory concentration (MIC) of *S.* Infantis strains was determined by means of the Sensititre System (Thermo Fisher Scientific, Waltham, MA, USA). In brief, a single *S.* Infantis colony was inoculated into 10 mL Brain Heart Infusion (BHI) broth and incubated overnight at 37 °C. The bacterial suspension was then diluted into demineralized water (Thermo Fisher Scientific, Waltham, USA) at the concentration of 0.5 Mc Farland. Ten mL of the diluted bacterial suspension were transferred into 11 mL of Cation-adjusted Mueller-Hinton broth (Thermo Fisher Scientific, Waltham, USA); then 50 µL were dispensed into a microwell plate and incubated overnight at 35 +/− 1 °C (EUVSEC, Thermo Fisher Scientific, Waltham, USA). Subsequently, the turbidity of the wells was evaluated by the Sensititre Vizion Digital MIC Viewing System (Thermo Fisher Scientific, Waltham, USA) through the Sensititre System software (Thermo Fisher Scientific, Waltham, USA) according to the EU Directive 2013/652/EU of 12 November 2013.

In the present study, the MIC value for twelve antibiotics was evaluated: ampicillin (AMP), cefotaxime (CEF), ceftazidime (CAZ), chloramphenicol (CLO), ciprofloxacin (CIP), colistin (COL), gentamicin (GEN), meropenem (MERO), nalidixic acid (NAL), tetracycline (TET), tigecycline (TGC), and trimethoprim (TMP). The *E. coli* strain ATCC 25922 (Thermo Fisher Scientific, Waltham, USA) was used as a control. The MIC values of the antimicrobial agents tested were recorded for each isolate and compared to breakpoints defined for *Enterobacterales* by the EUCAST (Table 5). In the evaluation of the results, strains displaying resistance to at least three antibiotic classes were considered multidrug-resistant (MDR) [30].

### 4.3. Molecular Assays

#### 4.3.1. DNA Extraction

One *S.* Infantis colony was suspended in 200 µL of molecular biology-grade water in a centrifuge tube and vortexed for a few seconds. The suspension was incubated at 95 °C for 15 min in a termomixer (Eppendorf) and then centrifuged for 10 min. (11,200 rcf). The quality (OD 260/280 = 1.8–2.0) of the DNA extracts was evaluated by utilizing a spectrometer (NanoDrop ONE^C^). Three μL of the template DNA were used in the PCR reactions.

#### 4.3.2. MLVA Assay

For the MLVA assay, in a thermocycler (CFX96^TM^ Real-time System—BioRad, Hercules, USA), five variable numbers of tandem repeats (VNTR) loci have been amplified in accordance with Kjeldsen et al. (2015) [31]. Out of these five loci, three (STTR3; STTR5; STTR9) were described in the *S*. Typhimurium genome [32], one (SG2) in *S*. Gallinarum [33], and one (Sty19) in *S*. Paratyphi A [34]. The locus name, primer sequence, and repeat size are shown in Table 6.

A triplex (STTR3; STTR5; STTR9) and a duplex (SG2; Sty19) PCR amplifications were carried out in a total volume of 25 µL in accordance with Kjeldsen et al. (2015) [32]. The Amplification reactions were performed in a T100 Thermal Cycler (Bio-Rad Laboratories). For the capillary electrophoresis assay, amplicons were diluted in sterile water (1:60), 1:l of the dilution was mixed with a solution (12:1) of Hi-Di formamide (Life Technologies) and GeneScan 500 LIZ internal size marker (Life Technologies). The solutions were then denatured (3’ at 96 °C), cooled for a few minutes, and finally analyzed in a SeqStudio Genetic Analyzer (Applied Biosistems Waltham, USA). The *S.* Gallinarum, *S.* Paratyphi, and *S.* Typhimurium strains provided by SSLCR were used as a control.

### 4.4. Clustering Analysis

Analysis was performed through RStudio software (version: 2022.12.0 Build 353). The dataset was made up of 185 statistical units and five variables which were all continuously quantitative. In order to make the variables comparable, the data were standardized. Standardization consisted of transforming each variable by subtracting the mean value and dividing by the standard deviation. A hierarchical cluster analysis was performed with Ward’s method, which measures the similarity of samples based on their Euclidean distance.

### 4.5. Statistical Analysis

The *AB test* was used to compare the frequency of isolation of *S*. Infantis from the considered sources. For the Z score (**Z**) evaluation, a confidence level of 0.95 was applied in the formula. Moreover, the discriminatory capability of each allele (allele discriminatory power—ADP) and the species diversity index were evaluated by means of the method proposed by Hunter (1990) [35] and Zaluga et al. (2013) [36], using a free discriminatory calculator (http://insilico.ehu.es/mini_tools/discriminatory_power/index.php accessed on 12 December 2022) and https://www.omnicalculator.com accessed on 12 December 2022). Species diversity was also assessed through the Shannon index calculation.

## 5. Conclusions

In conclusion, the data presented here compile an overview of the prevalence, distribution, and antibiotic resistance profile of *S.* Infantis strains isolated in the south of Italy. *S*. Infantis was strictly related to broiler sources. A high prevalence of resistant and MDR *S.* Infantis strains was found. Specifically, *S.* Infantis showed a worrying resistance against fluoroquinolones which are widely used for treatment against invasive salmonellosis in humans, and ampicillin and tetracycline, which are used in veterinary medicine as first-line treatment in animal infections. Therefore, more stringent control on the use of antimicrobials in veterinary medicine is indispensable. Moreover, in the present work, the use of the AMR profile and the MLVA profile, alone or in combination, was not sufficient to understand the complexity of the epidemiological relationships between *S.* Infantis strains, and therefore an alternative methodology is needed to investigate genetic similarities and differences among the strains isolated from different sources.

## Figures and Tables

**Table 1:** Number (N°), source, and sampling site of *Salmonella* Infantis strains (n = 185) isolated between 2018 and 2020.

Origin		N°	Source	N°	Sampling Site
Mammals	cow/calves	2	meat and meat products	2	retail market
	water buffalo	2	fecal swabs	2	farms
	pig	3	sponge	3	slaughterhouse
	wild boars	2	tissue/sponge	2	hygiene controls after hunting
	human	7	fecal swabs	5	hospital
			urine	1	
			blood	1	
poultry		162	poultry meat	26	retail market (butcher’s shop)
			surface swabs and sponges	14	farms (broiler)
			surface swabs and sponges	8	farms (laying hens)
			poultry products	78	retail market (large-scale distribution)
			eggs	19	retail market (large-scale distribution)
			neck skin samples	17	slaughterhouse
mussels		7	mussels	5	farms
			mussels	2	retail market
Total		185		185	

**Table 2:** Number and percentage of completely susceptible *S.* Infantis strains (S), isolates displaying intermediate resistance (I) or completely resistant (R) to Ampicillin (AMP), Cefotaxime (CEF), Ceftazidime (CAZ), Chloramphenicol (CLO), Ciprofloxacin (CIP), Colistin (COL), Gentamicin (GEN), Meropenem (MERO), Nalidixic acid (NAL), Tetracycline (TET), Tigecycline (TGC), and Trimethoprim (TMP) and originating from poultry, mussels, and humans. Isolates from cow/calves, water buffalo, pig, and wild boars were summed and are denoted as“Other mammals.

		AMP		CEF		CAZ		CLO		CIP		COL		GEN		MERO		NAL		TET		TGC		TMP	
Source		N.	%	N.	%	N.	%	N.	%	N.	%	N.	%	N.	%	N.	%	N.	%	N.	%	N.	%	N.	%
Poultry	R	95	58.64	54	33.33	34	20.99	74	45.68	91	56.17	3	1.85	1	0.62	1	0.62	157	96.91	124	76.54	2	1.23	126	77.78
	I	0	0	3	1.85	118	72.84	5	3.09	53	32.72	23	14.20	0	0	0	0	0	0	1	0.62	0	0	0	0
	S	67	41.36	105	64.81	10	6.17	83	51.23	18	11.11	136	83.95	161	99.38	161	99.38	5	3.09	37	22.84	160	98.77	36	22.22
Mussels	R	0	0	0	0	0	0	1	14	0	0	0	0	0	0	0	0	0	0	0	0	0	0	2	29
	I	0	0	0	0	0	0	0	0	0	0	2	29	0	0	0	0	0	0	0	0	0	0	0	0
	S	7	100	7	100	7	100	6	86	7	100	5	71	7	100	7	100	7	100	7	100	7	100	5	71
Humans	R	5	71.43	3	42.86	1	14.29	0	0	4	57.14	0	0	0	0	0	0	5	71.43	5	71.43	0	0.00	4	57.14
	I	0	0	0	0	2	28.57	0	0	1	14.29	0	0	0	0	0	0	0	0	0	0	0	0	0	0
	S	2	28.57	4	57.14	4	57.14	7	100.00	2	28.57	7	100.00	7	100.00	7	100.00	2	28.57	2	28.57	7	100.00	3	42.86
Other mammals	R	0	0	0	0	0	0	0	0	0	0	0	0	0	0	0	0	4	44.44	3	33.33	0	0	2	22.22
	I	0	0	0	0	0	0	0	0	4	44.44	0	0	0	0	0	0	0	0	0	0	0	0	0	0
	S	9	100	9	100	9	100	9	100	5	55.56	9	100	9	100	9	100	5	55.56	6	66.67	9	100	7	77.78

**Table 3:** Allele discriminatory power (ADP), Shannon diversity index, number of *S.* Infantis strains, size, and identity (ID) for each variable-number of tandem repeat (VNTR) locus.

VNTR							ADP			Shannon Diversity Index
Locus (n = 3)	Sty19									
Locus Size (bp)	56	66	69				0.24			0.48
Number of strains	160	9	16							
Locus ID	ST1	ST2	ST3							
Locus (n = 6)	SG2									
Locus Size (bp)	154	286	299	310	66	77	0.55			1.16
Number of strains	120	12	26	6	14	7				
Locus ID	CG1	CG2	CG3	CG4	CG5	CG6				
Locus (n = 5)	STTR3									
Locus Size (bp)	232	264	331	490	495		0.25			0.59
Number of strains	8	7	4	7	159					
Locus ID	SRA1	SRA2	SRA3	SRA4	SRA5					
Locus (n = 4)	STTR5									
Locus Size (bp)	286	297	303	309			0.53			1.01
Number of strains	32	120	20	13						
Locus ID	SRB1	SRB2	SRB3	SRB4						
Locus (n = 1)	STTR9									
Locus Size (bp)	154						0.0			0.0
Number of strains										
Locus ID	SRC1									

**Table 4:** Correlations between *S.* Infantis strains taking in consideration the cluster, geographical location (AV = Avellino; SA = Salerno; BN = Benevento; NA = Naples), source, antimicrobial resistance (AMR) profile, MLVA profile, date of sampling and sampling site.

Cluster	Province	Source	AMR Profile	MLVA	Date	Sampling Site
1	AV	wild boar	susceptible	56;154;286;66;495	19 February 2020	hunting house
	AV	wild boar	susceptible	56;154;286;66;495	7 April 2020	hunting house
2	SA	cow	TRIM;TET;NAL;CIP	56;154;297;66;495	6 June 2018	supermarket
	SA	cow	TRIM;TET;NAL;CIP	56;154;297;66;495	6 June 2018	supermarket
	BN	poultry	TRIM;NAL;CIP;CLO;CAZ;FOT;AMP	56;154;297;66;495	24 November 2020	supermarket
	NA	poultry	TRIM;NAL;CIP;CLO;CAZ;FOT;AMP	56;154;297;66;495	15 January 2020	supermarket
	BN	poultry	TRIM;NAL;CIP;CLO;CAZ;FOT;AMP	56;154;297;66;495	8 June 2021	supermarket
	BN	poultry	TRIM;NAL;CIP;CLO;CAZ;FOT;AMP	56;154;297;66;495	8 June 2021	supermarket
	BN	poultry	TRIM;NAL;CIP;CLO;CAZ;FOT;AMP	56;154;297;66;495	8 June 2021	supermarket
	NA	poultry	TRIM;NAL;CIP;CLO;CAZ;FOT;AMP	56;154;297;66;495	14 July 2020	slaughterhouse
	NA	poultry	TRIM;NAL;CIP;CLO;CAZ;FOT;AMP	56;154;297;66;495	15 July 2020	supermarket
	NA	poultry	TRIM;NAL;CIP;CLO;CAZ;FOT;AMP	56;154;297;66;495	11 October 2021	supermarket
4	SA	w buffalo	susceptible	69;154;297;153;232	25 March 2021	farm
	SA	w buffalo	susceptible	56;154;309;310;495	15 March 2019	farm
6	NA	poultry	TRIM;TET;NAL;CIP;AMP	56;154;292;66;493	14 January 2019	supermarket
	AV	human	TRIM;TET;NAL;CIP;AMP	66;154;297;153;495	4 November 2019	hospital
	SA	poultry	TET;NAL;CIP	69;154;309;66;495	29 July 2020	supermarket
	AV	human	TET;NAL;CIP	66;154;297;153;495	20 September 2020	hospital

**Table 5:** EUCAST breakpoint tables for interpretation of MICs of Enterobacterales.

Antibiotics	MIC Breakpoints (µg/mL)		
	Susceptible (S)	Intermediate (I)	Resistant (R)
Ampicillin (AMP)	<8	8	>8
Cefotaxime (CEF)	<0.5	0.5	>0.5
Ceftazidime (CAZ)	<2	2	>2
Chloramphenicol (CLO)	<16	16	>16
Ciprofloxacin (CIP)	<0.06	0.06	>0.06
Colistin (COL)	<2	2	>2
Gentamicin (GEN)	<2	2	>2
Meropenem (MERO)	<0.12	0.12	>0.12
Nalidixic Acid (NAL)	<16	16	>16
Tetracycline (TET)	<8	8	>8
Tigecycline (TGC)	<1	1	>1
Trimethoprim (TMP)	<2	2	>2

**Table 6:** Selected primers for the amplification of VNTRs.

VNTR	Size (bp)	Primers	References
STTR3	27/33	F- PET-CCCCCTAAGCCCGATAATGG	[32]
		R- TGACGCCGTTGCTGAAGGTAAT	
STTR5	6	F- VIC-ATGGCGAGGCGAGCAGCAG	[32]
		R- GGTCAGGCCGAATAGCAGGA	
STTR9	9	F- 6FAM-AGAGGCGCTGCGATTGACGA	[32]
		R- CATTTTCCACAGCGGCAGTTTTT	
SG2	8	F- NED-GTGATGATCATGGCGGACT	[33]
		R- CAGGTGGAACAGGAACTTC	
Sty19	9	F- 6FAM-CATCGTATTGTCAGGGTGGA	[34]
		R- TTCCCTGCGAGGAAAAGTT	

## Data Availability

The data presented in this study are available upon request from the corresponding author.

## Supplementary Materials

The following supporting information can be downloaded at: https://www.mdpi.com/article/10.3390/ijms24065492/s1.
