# Supplementary figures and images for: Antimicrobial Resistance and Genomic Characterization of Salmonella Infantis from Different Sources
Source: Int J Mol Sci. 2023 Mar 13;24(6):5492. doi: 10.3390/ijms24065492 (PMC10049457; doi:10.3390/ijms24065492)

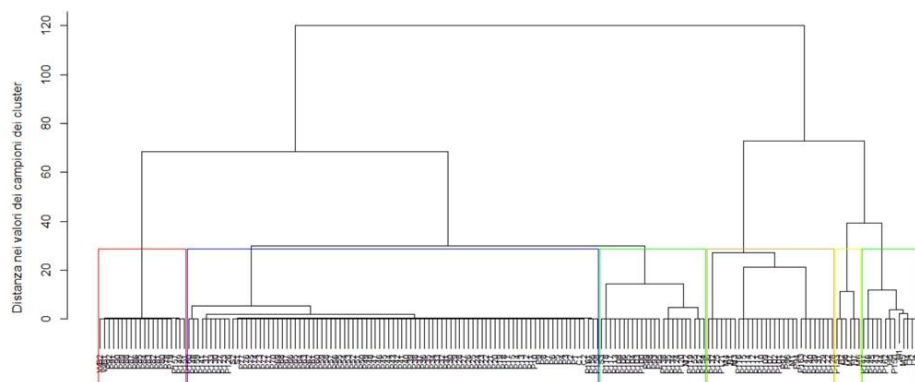

Figure S1: Dendrogram deduces from cluster analysis of the 185 *S. Infantis* strains.

Supplement: Supplementary file 1 [file ijms-24-05492-s001.zip › ijms-2279704-supplementary.pdf]
